# Characteristic Analysis on UAV-MIMO Channel Based on Normalized Correlation Matrix

**DOI:** 10.1155/2014/206185

**Published:** 2014-04-14

**Authors:** Gao Xi jun, Chen Zi li, Hu Yong Jiang

**Affiliations:** Unmanned Aerial Vehicle Department, Mechanical Engineering College, Shijiazhuang, Hebei 050003, China

## Abstract

Based on the three-dimensional GBSBCM (geometrically based double bounce cylinder model) channel model of MIMO for unmanned aerial vehicle (UAV), the simple form of UAV space-time-frequency channel correlation function which includes the LOS, SPE, and DIF components is presented. By the methods of channel matrix decomposition and coefficient normalization, the analytic formula of UAV-MIMO normalized correlation matrix is deduced. This formula can be used directly to analyze the condition number of UAV-MIMO channel matrix, the channel capacity, and other characteristic parameters. The simulation results show that this channel correlation matrix can be applied to describe the changes of UAV-MIMO channel characteristics under different parameter settings comprehensively. This analysis method provides a theoretical basis for improving the transmission performance of UAV-MIMO channel. The development of MIMO technology shows practical application value in the field of UAV communication.

## 1. Introduction

With the continuous development of UAV in the recent years, it is applied much more widely than ever before. The high-performance mission payload has also placed higher requirements on the communication capacity of UAV data link. However, the traditional UAV-SISO (single-input single-output) data link restrains the quick, accurate, and vast information exchange between UAV and the ground. As MIMO (multi-input multioutput) technology can improve the communication system capacity with increasing spectrum resource expensing and emission power [1], the application of MIMO technology in UAV data link is a new method to improve the communication capacity of UAV. The characteristics of UAV-MIMO channel influence the performance of UAV-MIMO data link to a great extent; therefore, the characteristic analysis on UAV-MIMO channel presents great significance in applying MIMO technology to UAV data link.

Research on the application of MIMO technology in aircraft communication system has been proposed in recent years [2–6]. Literature [2] has researched on the characteristics of the capacity of MIMO data link in aviation, but it only considered the diffuse function. Literature [3] has studied the problem of antenna being shielded by the fuselage in the maneuvering flight of aircraft and put forward a scheme which could realize the reliable communication between aircraft and the ground by using multiple antennas and space-time coding technology, but its performance analysis was based on the white Gaussian noise channel without considering the modeling analysis on the aeronautical communication channel. Literature [4] presented the method of applying the combination of beam forming and differential space-time modulation technique to aeronautical communication and the angle of arrival and BER performance have been analyzed, but it also did not take the modeling analysis on the aeronautical channel into consideration. Literature [5] has made allowance for the spatial correlation and the shielded antenna and put forward the parametric statistical model of the multiantenna communication channel of an unmanned helicopter, but the model failed to consider the influence of the antenna array structure at the transmitting and receiving parts. Literature [6] established the GBSBCCS (geometrically based single bounce concentric cylinder scattering) wideband transmitting model, but it just made analysis on the correlation of UAV-MIMO channel diffuse transmitting model.

According to the characteristics of the actual communication environment of UAV, in this paper, it mainly studies the downlink channel of UAV data link and establishes a three-dimensional GBSBCM UAV-MIMO channel model. Based on the method of channel matrix decomposition and channel coefficient normalization, the formula of UAV-MIMO normalized average channel correlation matrix is deduced. Through the analysis on the channel capacity and the condition number of matrix as well as other characteristic parameters, comparative analysis on the changes of channel characteristics under different parameters has been made, namely, the different antenna layouts, different transmission distance, different positions of diffuser, and so on. In the analysis, both the UAV and the ground control station are in the two-element antenna array models, and the analysis results can be extended to any multielement antenna array.

The layout of this paper is as below. Section 2 establishes the three-dimensional GBSBCM channel model of UAV-MIMO with LOS, SPE, and DIF components; Section 3 describes the characteristic parameters of MIMO channel; Section 4 presents the simple form of space-time-frequency channel correlation function and the expression of normalized channel correlation matrix is deduced; Section 5 makes simulation analysis on the characteristics of UAV-MIMO channel; Section 6 presents the conclusion.

## 2. UAV-MIMO Channel Model

**Figure 1:**
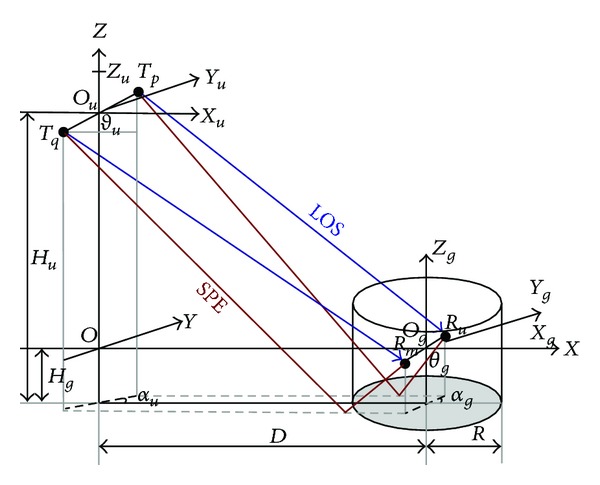
Communication model of LOS and SPE components.

**Figure 2:**
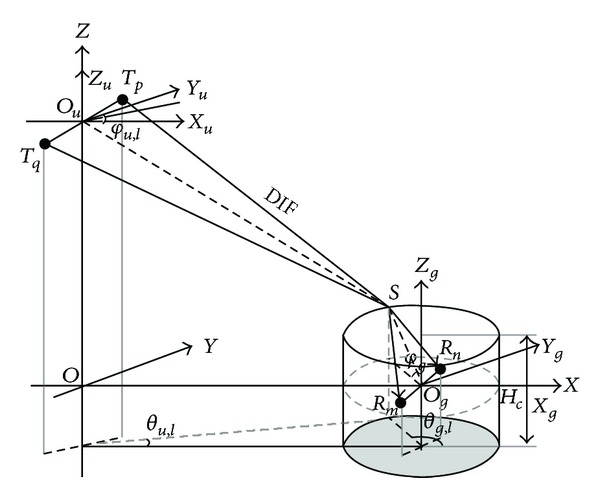
Communication model of DIF component.

The traditional aeronautical MIMO channel is mainly divided into LOS (line-of-sight) and SPE (specular) components [2, 7], but UAV communication channel includes strong LOS and SPE components as well as some DIF (diffuse) component [8]. Therefore, aeronautical communication channel cannot be used to characterize UAV-MIMO channel perfectly. The in-flight UAV is far from the ground control station and a big height difference exists in between; the ground control station is surrounded by diffusers, so that it can be considered to be in the center of diffuse environment. As the height of the ground control station is much lower than that of the surrounding mountains or large buildings as well as other diffusers, the existence of pitching angle cannot be ignored. According to the research of literature [9–13], if an obvious height difference exists between the transmitting station and the receiving station, diffusers are distributed around the receiving station and the pitching angle exists; “circular” diffuse model can be used to describe the statistical characteristics of the channel. Relevant measurement results have also proved the reasonability [12, 13] of the circular distribution of diffuser. Therefore, combined with the characteristics of UAV communication environment, the UAV-MIMO transmission model with LOS, SPE, and DIF components which is constructed based on GBSBCM is shown Figures 1 and 2.

Assume that the diffusers (height is *H*_*c*_) are uniformly distributed in the circle which regards the midpoint of the ligature of the receiving antennas (height is *H*_*g*_) as the center with a radius of *R*; the flight height of UAV is *H*_*u*_ and the horizontal flight distance is *D*. Define the coordinate system as follows. On plane *X* − *Y*, the tangent circle regards the midpoint *O*_*g*_ of the ligature of the two antennas of the receiving station as the center; the projection of the center *O*_*u*_ of UAV circular array antenna on plane *X* − *Y* is *O* which is the origin of the coordinate system; connect *O* − *O*_*g*_ as the axis *X* and *O* − *O*_*u*_ as the axis *z*; UAV coordinate axis *Z*_*u*_ coincides with axis *Z*, plane *X*_*u*_ − *Y*_*u*_ is parallel to the plane *X* − *Y*; axis *X*_*g*_ and axis *x* of the receiving coordinate system coincide with each other; plane *Y*_*g*_ − *Z*_*g*_ is parallel to plane *Y* − *Z*; thus the transmitting coordinate system *O*_*u*_ − *X*_*u*_ *Y*_*u*_ *Z*_*u*_, receiving coordinate syst *O*_*g*_ − *X*_*g*_ *Y*_*g*_ *Z*_*g*_ and coordinate *O* − *XYZ* all have the same properties of parallel. In this coordinate system, the pitching angle and the azimuth angle of the ligature of UAV transmitting antennas are *ϑ*_*u*_ and *α*_*u*_, respectively. Those of the ligature of the ground receiving antennas are *ϑ*_*g*_ and *α*_*g*_, respectively. *s*_*l*_ is the number *l* diffuser; the pitching angle and the azimuth angle of diffuse component from UAV are *φ*_*u*,*l*_ and *θ*_*u*,*l*_. After being diffused, it reaches the ground station with the pitching angle and azimuth angle of *φ*_*g*,*l*_ and *θ*_*g*,*l*_, respectively.

The model not only considers the flight height, the horizontal flight distance, and the attitude angle of UAV as well as other flight parameters, in the height of ground receiving station, the angle of receiving antennas, and the diffuse radius and height as well as other diffuse parameters of the environment but also considers the various components of the channel, making it consistent with the real environment to a great extent. This model not only meets the application requirements but also reflects the characteristics of UAV-MIMO channel.

## 3. Characteristic Parameters of MIMO Channel

### 3.1. Condition Number

The condition number of channel matrix can reflect the correlation between the condition of MIMO channel matrix and the component channel, which is defined as the ratio of the maximum and minimum characteristic values of channel matrix **H** and can be expressed as [14]

(1) $$\begin{array}{c} \text{Condition}\left(H\right)=\frac{\lambda_{\max}\left(H\right)}{\lambda_{\min}\left(H\right)}. \end{array}$$

In the MIMO system, its meaning is the closer the condition number approaches to 1, the better the transmitting conditions of each parallel component channels and the quality of MIMO channel will be; the larger the ratio is, the poorer the quality of the channel will be. Therefore, through analyzing the condition number of MIMO channel matrix, we can get better understanding of the channel.

### 3.2. Channel Capacity

According to the Shannon theory, if the channel of the transmitting station is unknown and the channel coefficient is fixed, the channel capacity of MIMO system with *n*_*T*_ transmitting antennas and *n*_*R*_ receiving antennas can be expressed as [1, 2]

(2) $$\begin{array}{c} C=\text{lo}{\text{g}}_{2}\left[\det\left(I_{n_{R}}+\frac{\text{SNR}}{n_{T}}HH^{*}\right)\right]\left(\text{bit}/\text{s}/\text{Hz}\right), \end{array}$$

where SNR is the signal-to-noise ratio; **H** is the channel correlation matrix of *n*_*T*_ × *n*_*R*_; H* is the conjugate transpose of **H**. If the channel coefficient is a random variable, the channel capacity mentioned above is the instantaneous channel capacity. Then, the ergodic capacity can be used to describe the channel capacity, namely, acquire the average channel capacity by calculating the average value of all the channel coefficients, which is expressed as

(3) $$\begin{array}{c} \bar{C}=E_{H}\left(\text{lo}{\text{g}}_{2}\left[\det\left(I_{n_{R}}+\frac{\text{SNR}}{n_{T}}HH^{*}\right)\right]\right)\left(\text{bit}/\text{s}/\text{Hz}\right). \end{array}$$

## 4. Normalized Correlation Matrix of UAV-MIMO Channel

The time-frequency channel coefficients of the LOS, SPE, and DIF components of UAV transmitting antenna-ground receiving antenna component channel *h*_*np*_(*t*, *f*) can be expressed as

(4) $$\begin{array}{c} h_{\text{LOS}}^{}\left(t,f\right)=\eta_{\text{LOS}}^{}a_{\text{LOS}}^{}g_{\text{LOS}}^{}\left(t\right)e^{-j2\pi f\tau_{\text{LOS}}^{}},\\ h_{\text{SPE}}^{}\left(t,f\right)=\eta_{\text{SPE}}^{}a_{\text{SPE}}^{}g_{\text{SPE}}^{}\left(t\right)e^{-j2\pi f\tau_{\text{SPE}}^{}},\\ h_{\text{DIF}}^{}\left(t,f\right)=\eta_{\text{DIF}}^{}\underset{L\to \infty }{\lim}\frac{1}{\sqrt{L}}\overset{L}{\underset{l=1}{\sum }}g_{l}a_{\text{DIF}}^{}g_{\text{DIF}}^{}\left(t\right)e^{-j2\pi f\tau_{l}}. \end{array}$$

In these expressions, *η*, *a*, *τ*, and *g*(*t*) represent the scaling factor, pass loss, and pass delay of the LOS, SPE, and DIF components of the transmitting antenna-receiving antenna channel in the total received power as well as the function of phase deviation caused by the Doppler frequency shift due to the movement of transmitting path, the transmitting station, and the receiving station, respectively. *g*_*l*_ represents the stochastic gain caused by the number *l* diffuser between the transmitting antennas and the receiving antennas.

According to the expressions (1)~(3), the key part of the characteristic analysis on MIMO channel is to obtain the channel correlation matrix **H**. From expressions (4), we can see that the UAV channel coefficients are random variables, and thus the average channel characteristics can be obtained by calculating the average channel correlation matrix.

### 4.1. Space-Time-Frequency Correlation Function

In the condition of WSSUS (wide sense stationary uncorrelated scattering), assume that the probability density functions of the pitching angle and azimuth angle of the ground receiving antenna diffuse obey Von-Mises [15] distribution model and composite parameter [16] model. Associated with the UAV-MIMO channel model, take the transmission channel between the transmitting antenna *T*_*p*_ and *T*_*q*_ and the receiving antenna *R*_*n*_ and *R*_*m*_ as the example, the space-time-frequency correlation function of the LOS and SPE components can be simplified as

(5) Rnp,mqLOS(Δt,Δf) =E(hnp,LOS(t,f)hmq,LOS∗(t+Δt,f+Δf)) =ejk0(dnpLOS−dmqLOS)×RLOSejfLOS(Δt,Δf),

(6) Rnp,mqSPE(Δt,Δf) =E(hnp,SPE(t,f)hmq,SPE∗(t+Δt,f+Δf)) =ejk0{dnpSPE−dmqSPE}×RSPEejfSPE(Δt,Δf),

where

(7) dnpLOS=(((Hu+δpq2sinϑu)−(Hg+δnm2sinϑg))2+(D−δpq2cos⁡ϑucos⁡αu+δnm2cos⁡ϑgcos⁡αg)2)1/2,dmqLOS=(((Hu−δpq2sinϑu)−(Hg−δnm2sinϑg))2+(D+δpq2cos⁡ϑucos⁡αu−δnm2cos⁡ϑgcos⁡αg)2)1/2,dnpSPE=(((Hu+δpq2sinϑu)+(Hg+δnm2sinϑg))2+(D−δpq2cos⁡ϑucos⁡αu+δnm2cos⁡ϑgcos⁡αg)2)1/2,dmqSPE=(((Hu−δpq2sinϑu)+(Hg−δnm2ssinϑg))2+(D+δpq2cos⁡ϑucos⁡αu−δnm2cos⁡ϑgcos⁡αg)2)1/2.

Among them, *k*_0_ = 2*π*/*λ* is free space wave number and *λ* is the free space wavelength. *δ*_*pq*_ and *δ*_*nm*_ represent the spacing between transmitting antennas and receiving antennas, respectively; *R*_LOS_ and *R*_SPE_ are the ranges of LOS and SPE correlation functions; *f*_LOS_(Δ*t*, Δ*f*) is the function of phase change caused by time and frequency change; Δ*t* and Δ*f* are the functions of variables, which conforms to *f*_LOS_(0,0) = *f*_SPE_(0,0) = 0.

According to the space-time-frequency correlation function of DIF mentioned in the literature [6], the space-time-frequency correlation function of DIF component can be further simplified as

(8) Rnp,mqDIF(Δt,Δf) =RDIFejfDIF(Δt,Δf)×ej2πk0(cos⁡⁡ϑucos⁡⁡αu−Δ1sin⁡ϑu)/1+Δ12  ×I0(x2+y2)×R(m,n)I0(k),

where

(4.1) x=jk0δnmcos⁡ϑgcos⁡αg+kcos⁡θg0+fx(Δt,Δf),y=jk0(δnmcos⁡ϑgsinαg+δpqcos⁡ϑusinαuΔxy1+Δ12) +ksinθg0+fy(Δt,Δf),Δxy=RD,  Δ1=(Hu−Hg−Rtgφg,l)D,R(m,n)=∫−arctg(Hg/R)arctg((HC−Hg)/R)ejk0δnmφgsinϑgf(φg,l)dφg,l.

*I*_0_(·) represents the zero-order Bessel modified function of the first kind; *k* is the angle spread factor of Von-Mises distribution; *θ*_*g*0_ is the mean value of the azimuth spread of ground receiving station of DIF; *f*(*φ*_*g*,*l*_) is consistent with composite parameter distribution model; *R*_DIF_ is the range of DIF correlation function; *f*_DIF_(Δ*t*, Δ*f*), *f*_*x*_(Δ*t*, Δ*f*),and *f*_*y*_(Δ*t*, Δ*f*) are also the functions varying with Δ*t* and Δ*f*, which meets the condition of

(10) $$\begin{array}{c} f_{\text{DIF}}\left(0,0\right)=f_{x}\left(0,0\right)=f_{y}\left(0,0\right)=0. \end{array}$$

### 4.2. Channel Matrix Decomposition

UAV-MIMO channel correlation matrix **H** can be decomposed into

(11) $$\begin{array}{c} H=\eta_{\text{LOS}}H_{\text{LOS}}+\eta_{\text{SPE}}H_{\text{SPE}}+\eta_{\text{DIF}}H_{\text{DIF}}. \end{array}$$

In this part, **H**_LOS_, **H**_SPE_, and **H**_DIF_ represent the average channel correlation matrix of LOS, SPE, and DIF components, respectively; *η*_LOS_, *η*_SPE_, and *η*_DIF_ represent the scaling factor of LOS, SPE, and DIF components in the total received power, respectively, which are expressed as

(12) ηLOS=KRice1+KRice+KRiceΓ2,ηSPE=ΓKRice1+KRice+KRiceΓ2,ηDIF=11+KRice+KRiceΓ2.

In this part, Γ ∈ [−1,1] is the SPE coefficient, namely, the ratio of the incident wave and specular wave; *K*_Rice_ ∈ [0, +*∞*) is Rice factor, namely, the ratio of power values of LOS component and DIF component.

In the UAV two-element array MIMO antenna system, the average channel correlation matrix of LOS component **H**_LOS_ can be expressed as

(13) $$\begin{array}{c} H_{\text{LOS}}=E\left\{\left[\begin{array}{cc} h_{np,\text{LOS}}\left(t,f\right) & h_{nq,\text{LOS}}\left(t,f\right)\\ h_{mp,\text{LOS}}\left(t,f\right) & h_{mq,\text{LOS}}\left(t,f\right) \end{array}\right]\right\}. \end{array}$$

In the expression, *h*_*np*,LOS_(*t*, *f*), *h*_*nq*,LOS_(*t*, *f*), *h*_*mp*,LOS_(*t*, *f*), and *h*_*mq*,LOS_(*t*, *f*) represent the channel coefficient of the LOS transmitting component from the transmitting antenna to the receiving antenna, respectively. The expressions **H**_SPE_ and **H**_DIF_ are similar to that of **H**_LOS_; just replace the channel coefficient of the matrix by the channel coefficients of reflection and scattering components.

### 4.3. Normalization of Channel Matrix

Take the LOS average channel correlation matrix as an example and solve the problem by using the method of the channel matrix normalization. The average channel correlation matrix of SPE and DIF components **H**_SPE_ and **H**_DIF_ can be calculated in the same way. The average channel correlation matrix of LOS component **H**_LOS_ is calculated in the following way: take *h*_*np*_^LOS^(*t*, *f*) as the benchmark, set *h*_*np*_^LOS^(*t*, *f*) = 1, and divide **H**_LOS_ by *h*_*np*_^LOS^(*t*, *f*), to preserve the correlation between channels and the expression of correlation matrix is deduced. Consider

(14) $$\begin{array}{c} H_{\text{LOS}}=E\left\{\left[\begin{array}{cc} 1 & \frac{h_{nq}^{\text{LOS}}\left(t,f\right)}{h_{np}^{\text{LOS}}\left(t,f\right)}\\ \frac{h_{mp}^{\text{LOS}}\left(t,f\right)}{h_{np}^{\text{LOS}}\left(t,f\right)} & \frac{h_{mq}^{\text{LOS}}\left(t,f\right)}{h_{np}^{\text{LOS}}\left(t,f\right)} \end{array}\right]\right\}. \end{array}$$

In formula (14), for example, *E*{*h*_*nq*_^LOS^(*t*, *f*)/*h*_*np*_^LOS^(*t*, *f*)} can be calculated as

(15) E{hnqLOS(t,f)hnpLOS(t,f)}=Rnp,nqLOS(0,0)Re{Rnp,nqLOS(0,0)}.

*R*_*np*,*nq*_^LOS^ is the channel correlation function when *δ*_*nm*_ = 0 in formula (5); similarly, *E*{*h*_*mp*_^LOS^(*t*, *f*)/*h*_*np*_^LOS^(*t*, *f*)} is solved by correlation function *R*_*np*,*nq*_^LOS^ while *R*_*mp*,*np*_^LOS^ is the channel correlation function of formula (5) when *δ*_*pq*_ = 0. Accordingly, we can get the average channel correlation matrix **H**_LOS_ of the LOS component.

To sum up, this method is to divide the channel matrix into the LOS, SPE, and DIF components, and then solve the problem by the normalizing each component, so as to get the required UAV-MIMO normalized channel correlation matrix **H**.

## 5. Characteristic Analysis on UAV-MIMO Channel

Based on the calculation method of UAV-MIMO normalized channel correlation matrix, we can make direct analysis on the characteristics of UAV-MIMO channel. This paper mainly analyzes the influence of UAV multiantenna layout, flight distance, and the position of diffuser as well as other parameters on the condition number of UAV-MIMO channel matrix and channel capacity. In the simulation, assume that the flight height of UAV *H*_*u*_ = 2 Km, horizontal flight distance *D* = 50 Km, the height of the antenna of ground station *H*_*g*_ = 5 m, the height of the diffuser *H*_*C*_ = 300 m, radius of diffuser (from diffuser to receiving antenna) *R* = 3 Km, spread average of azimuth angle  *θ*_*g*0_ = *π*/8, angle spread factor *k* = 0, interval of the transmitting antenna and receiving antenna is 10 times of wavelength, azimuth angle and pitching angle of transmitting antennas and receiving antennas are *π*/4, Rice factor *K*_Rice_ = 6 dB; reflectance Γ = −1, and receiving signal-to-noise ratio SNR = 16 dB.

### 5.1. Antenna Layout

**Figure 3:**
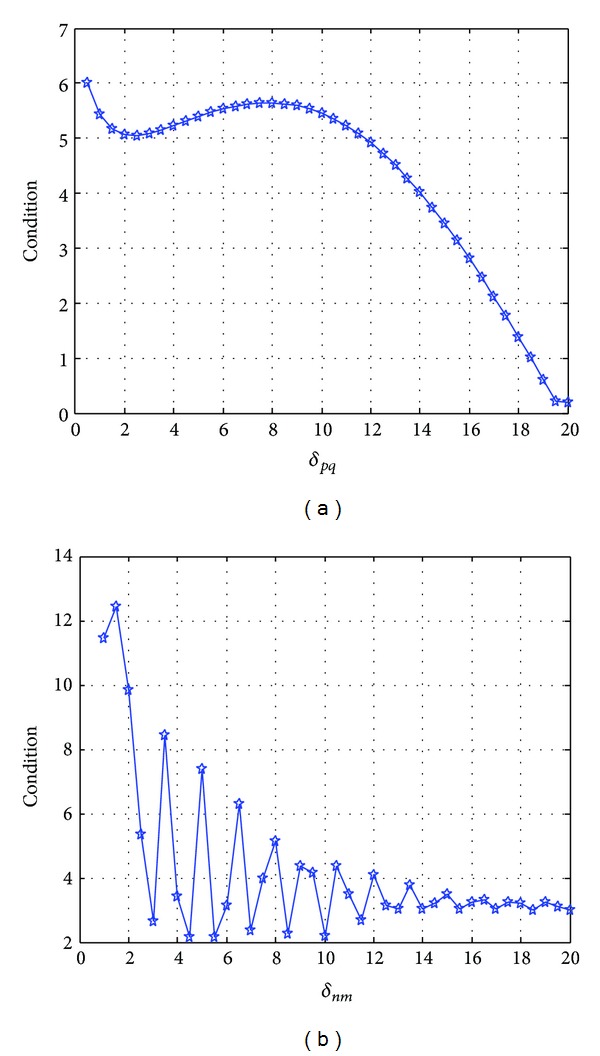
Influence of antenna interval on channel condition number. (a) Transmitting antenna; (b) receiving antenna.

Based on the analysis on the influence of antenna layout on UAV-MIMO channel characteristics, we can improve the performance of UAV-MIMO communication through reasonable layout. If the other simulation conditions keep unchanged, the influence of the change of transmitting interval on the condition number of the channel matrix is shown in Figure 3.  Figure 3(a) shows that when the interval of transmitting antenna increases, the condition number of channel matrix will decrease gradually; namely, the channel transmission quality will be improved.  Figure 3(b) shows that, along with the increase of the interval of receiving antenna, the condition number of channel matrix will decrease gradually; namely, increasing the interval of the receiving antenna of ground station can also improve UAV-MIMO channel transmission quality. If we set the condition number of channel matrix lower than 5 dB as the index, the interval of UAV aeronautical antenna will be not less than 10 times of wavelength and the interval of ground receiving antenna will be not less than 8 times of wavelength.

**Figure 4:**
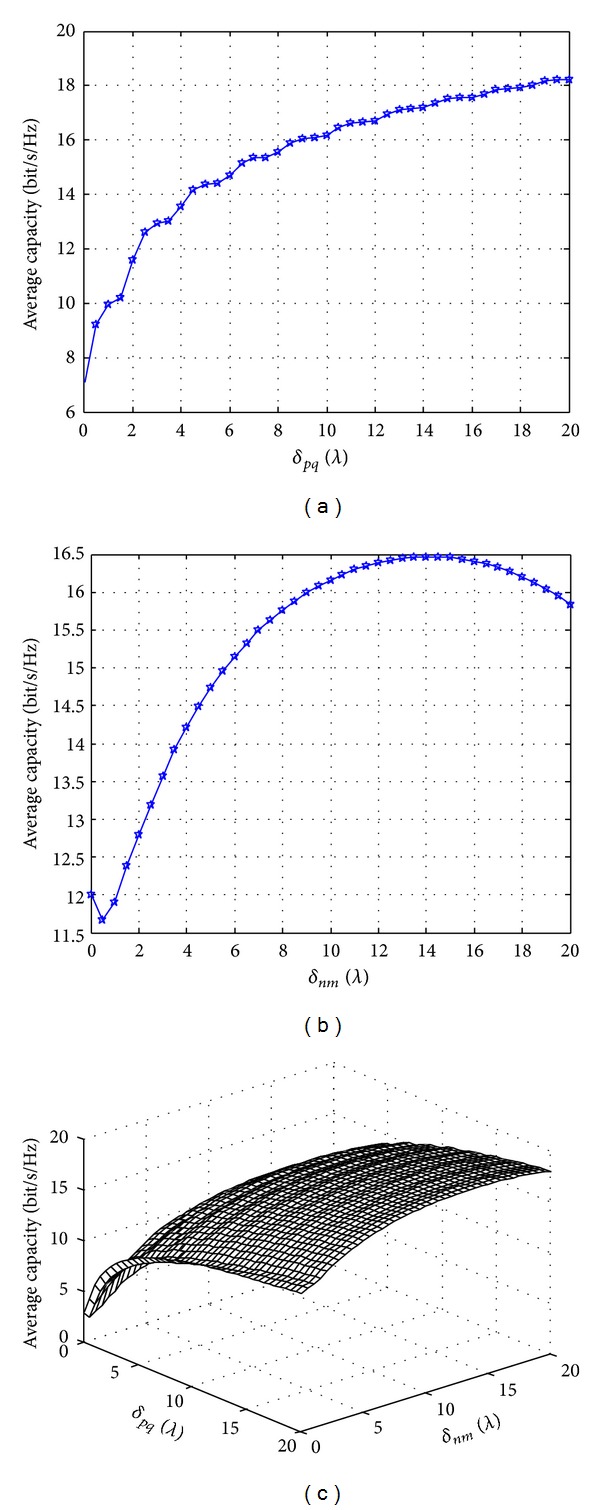
Influence of antenna interval on average channel capacity. (a) Transmitting antenna; (b) receiving antenna; (c) three-dimensional relation.

The relationship between the interval of transmitting and receiving antennas and the average channel capacity of UAV is shown in Figure 4.  Figure 4(a) shows the influence of transmitting antenna on average channel capacity of UAV when *δ*_*nm*_ = 10*λ*; when *δ*_*pq*_ < 10*λ*, the channel capacity is in linear growth; and when *δ*_*pq*_ ≥ 10*λ*, the growth of channel capacity is relatively slow, so we can set the interval of transmitting antenna as about 10*λ* according to the space of the UAV; Figure 4(b) shows the influence of receiving antenna on the average channel capacity of UAV when *δ*_*pq*_ = 10*λ*; along with the increase of the ground receiving antenna, the average channel capacity will increase because the increase of antenna interval reduces the correlation of channels in space but enhances the channel capacity; Figure 4(c) shows the three-dimensional relation of transmitting antenna, receiving antenna, and the average channel capacity of UAV; when the interval of ground receiving antenna is large, we can increase the channel capacity of UAV through reducing the interval of transmitting antenna, so as to effectively reduce the dependence of the antenna interval on the spatial structure of UAV.

**Figure 5:**
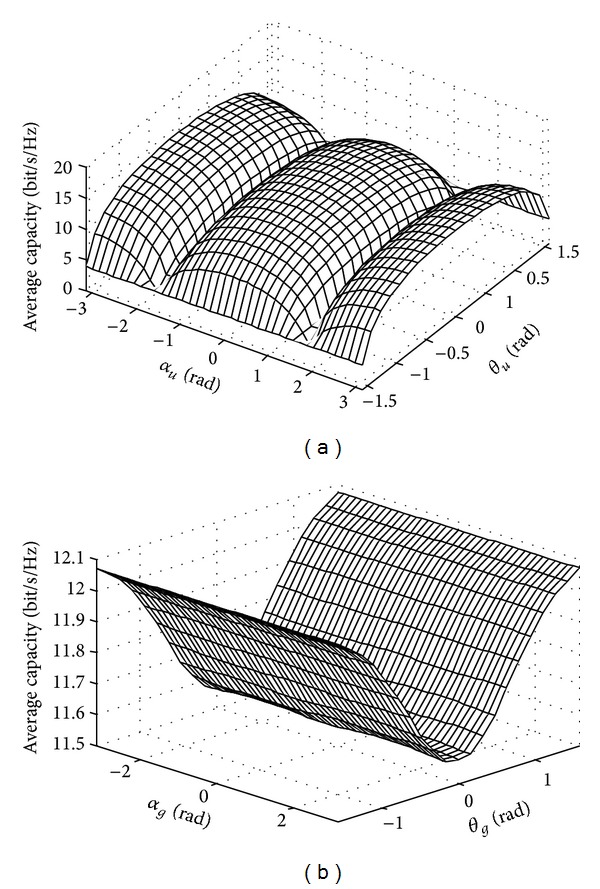
Influence of the position of antenna on the average channel capacity. (a) Transmitting antenna; (b) receiving antenna.

Besides the antenna interval, the azimuth angle and pitching angle of antenna and coordinate system also have great influence on the average channel capacity of UAV. If the other simulation conditions keep unchanged, the influence of the change of the antenna position on the average channel capacity of UAV is shown in Figure 5. From the influence of the pitching angle and azimuth angle of UAV antenna on the average channel capacity shown in Figure 5(a), we can see that the average channel capacity of UAV is symmetrical as the pitching angle and the azimuth angle are ranging between [−*π*/2 *π*/2]; the smaller the absolute value of the pitching angle is, the larger the average channel capacity will be; when the azimuth angle is ranging between [0,   ± *π*], the average channel capacity reaches the maximum value, so we can place the UAV antenna horizontally to get a larger channel capacity. From the influence of the pitching angle and azimuth angle of ground antenna on average channel capacity shown in Figure 5(b), we can see that the average channel capacity is symmetrical as the pitching angle that is ranging between [−*π*/2 *π*/2]; the smaller the absolute value of the pitching angle is, the larger the average channel capacity will be; and the influence of azimuth angle on the average channel capacity can be ignored, so we can place the ground antenna vertically to get a larger average channel capacity.

### 5.2. Flight Distance

**Figure 6:**
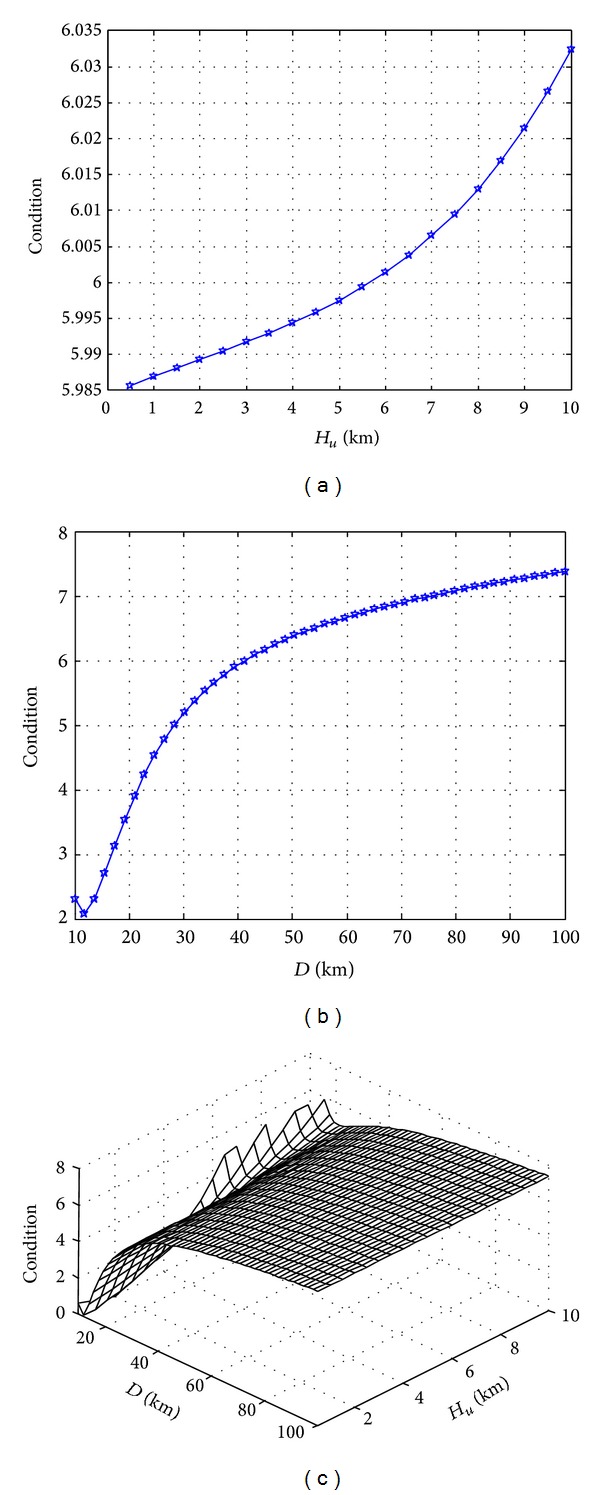
Influence of flight distance on the condition number of channel matrix. (a) Flight height; (b) flight distance; (c) three-dimensional relation.

In the process of UAV communication, the flight distance of UAV will influence the effect of communication, so it is necessary to analyze the influence of flight distance on the channel characteristics. If the other simulation conditions keep unchanged, the influence of the change of UAV flight distance on the condition number of the channel matrix is shown in Figure 6.  Figure 6(a) shows that, when the horizontal flight distance *D* = 50 Km, the higher the flight height of UAV is, the more the condition number of the channel matrix will be and the lower the transmission performance will be; Figure 6(b) shows that when *H*_*u*_ = 2 Km, the longer the flight distance of UAV is, the more the condition number of the channel matrix will be, and, similarly, the lower the transmission performance will be. If we set the condition number of channel matrix less than 6 dB as the index, the horizontal flight distance should be within 50 Km; Figure 6(c) shows the three-dimensional relationship of UAV flight height, horizontal flight distance, and the condition number of channel matrix. In realizing the same transmission performance of MIMO channel, we can reduce the flight height to achieve longer flight distance.

**Figure 7:**
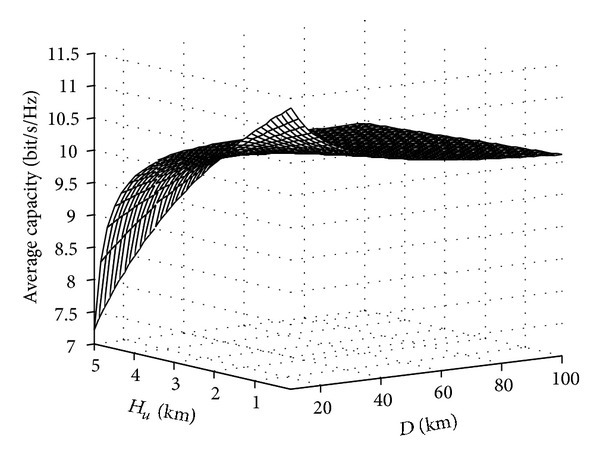
Influence of flight distance on the average channel capacity.

The influence of flight distance on the average channel capacity is simulated in Figure 7, which shows that the average channel capacity will be reduced if the flight distance is increasing. The reason is that the father flight distance reduces the spatial resolution of transmitting signal. So the UAV MIMO is the same with close quarters communications.

### 5.3. Parameters of Scatters

**Figure 8:**
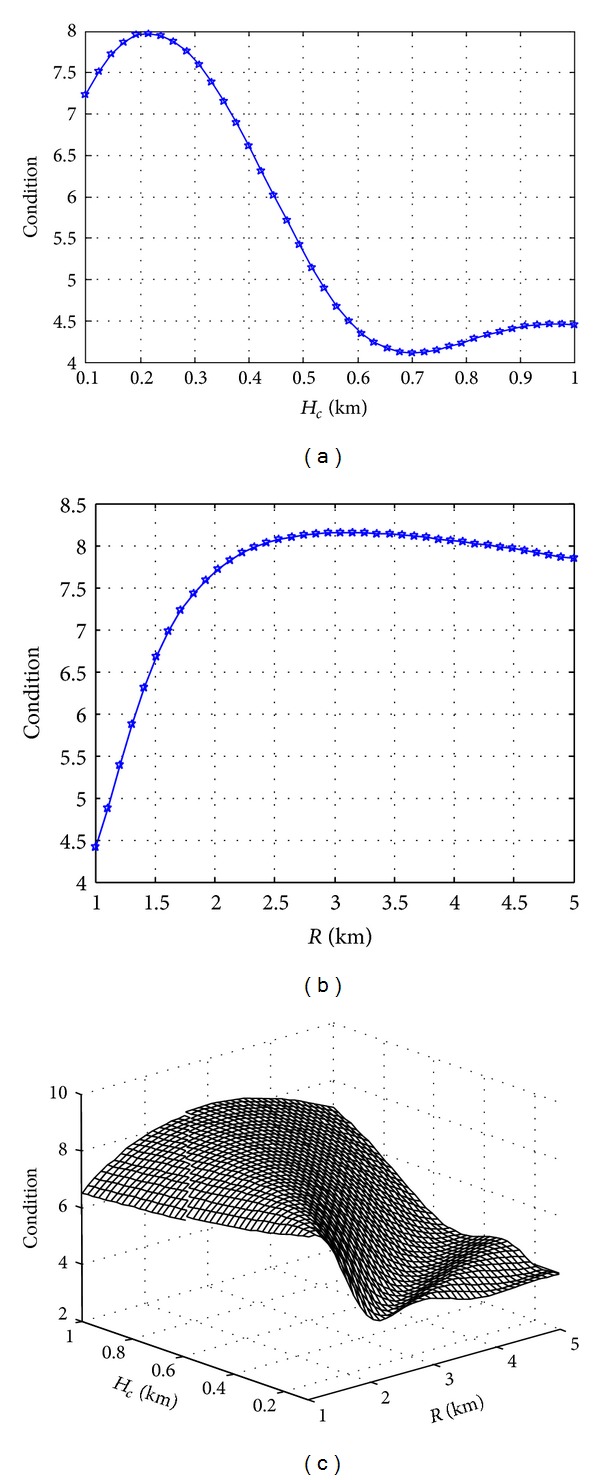
Influence of the diffuser parameters on the condition number of channel matrix. (a) Height of diffuser; (b) radius of diffuser; (c) three-dimensional relation.

In UAV communication model, the diffuser increases the multipath components, so the parameters of diffuser influence the UAV-MIMO communication performance. If the other simulation conditions keep unchanged, the influence of the change of diffuser parameters on the condition number of channel matrix is shown in Figure 8.  Figure 8(a) shows that, when the distance from diffusers to the ground station (radius of diffuser) *R* = 3 Km, the higher the height of diffuser is, the more the DIF multipath components will be and the smaller the condition number of channel matrix will be, so the transmission performance of the channel will be improved; Figure 8(b) shows that, when the height of diffuser *H*_*C*_ = 300 m, the larger the radius of diffuser is, the fewer the diffuse multipath components will be and the more the condition number of channel matrix will be, so the transmission performance of the channel will be degraded; Figure 8(c) shows the three-dimensional relation of the height of diffuser, the radius of diffuser, and condition number of channel matrix. Among the diffusers within a certain height, we can select the site as close as possible to the position of diffuser to reduce the condition number of the channel matrix, so as to improve the transmission performance of MIMO channel.

**Figure 9:**
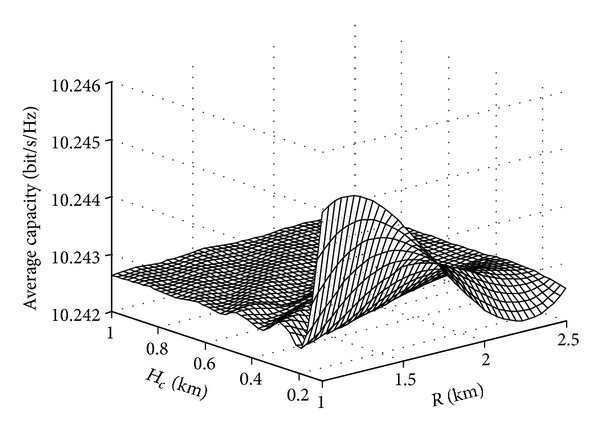
Influences of diffuser parameters on the average channel capacity.

The influence of diffuser parameters on the average channel capacity of UAV is shown in Figure 9. We can see that, in the same interval of antenna, the larger the DIF radius is, the lower the average channel capacity will be. That is to say, the more open the ground is and the further away it is from the diffuser, the weaker the spatial multipath resolution power will be and the smaller the average channel capacity will be; similarly, the higher the diffuser is, the stronger the spatial multipath resolution power will be and the larger the average channel capacity will be.

## 6. Conclusion

This paper constructs a three-dimensional GBSBCM channel model of UAV-MIMO. Based on the transmission model, it adopts the method of channel matrix decomposition and normalization to deduce the UAV-MIMO average channel correlation matrix and directly analyzes the influence of UAV multiantenna layout, flight distance, and the position of diffuser and other parameters on the characteristics of UAV-MIMO channel. The simulation results show that, through reasonably arranging the antenna internal and position and adjusting the flight distance of UAV and the site selection of the ground station, the transmission performance of UAV-MIMO channel can be improved, which lays a theoretical foundation for realizing the application of MIMO technology in UAV communication system.
